# Crosstalk of Histone and RNA Modifications Identified a Stromal-Activated Subtype with Poor Survival and Resistance to Immunotherapy in Gastric Cancer

**DOI:** 10.3389/fphar.2022.868830

**Published:** 2022-05-05

**Authors:** Cheng Yuan, Junchang Zhang, Cuncan Deng, Yujian Xia, Bo Li, Sijun Meng, Xinghan Jin, Lvjia Cheng, Huafu Li, Changhua Zhang, Yulong He

**Affiliations:** ^1^ Digestive Diseases Center, The Seventh Affiliated Hospital of Sun Yat-sen University, Shenzhen, China; ^2^ Guangdong Provincial Key Laboratory of Digestive Cancer Research, The Seventh Affiliated Hospital of Sun Yat-sen University, Shenzhen, China; ^3^ Department of Gastrointestinal Surgery, The First Affiliated Hospital of Sun Yat-sen University, Guangzhou, China; ^4^ Department of Thyroid Surgery, The Second Affiliated Hospital, College of Medicine, Zhejiang University, Hangzhou, China; ^5^ Scientific Research Center, The Seventh Affiliated Hospital of Sun Yat-sen University, Shenzhen, China; ^6^ Gastrointestinal Surgery, The First Affiliated Hospital of Jinan University, Guangzhou, China; ^7^ The Institute of Cancer Research, London, United Kingdom

**Keywords:** gastric cancer, histone modification, m6A, tumor microenvironment, prognosis, immunotherapy

## Abstract

Emerging evidence has revealed the pivotal role of epigenetic modifications in shaping the tumor microenvironment (TME). However, crosstalk between different modification types and their clinical relevance in cancers remain largely unexplored. In this study, using ChIP/MeRIP-seq data of seven human gastric cell lines, we systematically characterized the crosstalk of four epigenetic modification types including H3K4me1, H3K4me3, H3K27ac, and N6-methyladenosine (m6A) and identified a recurrent subtype with high FTO expression and low HDAC1 expression across three independent gastric cancer (GC) cohorts, which we named the epigenetic-modification-dysregulated (EMD) subtype. Patients of the EMD subtype were featured with poor survival, stromal activation, and immune suppression. Extensive relevance to clinical characteristics was observed in the EMD subtype, including the Lauren classification, MSI status, histological grade, TNM stage, the Asian Cancer Research Group classification, and the immune/fibrotic classification. An EMD score was then constructed using WGCNA and ssGSEA algorithms, to precisely recognize the EMD subtype and indicate prognosis and response to immunotherapy in multiple independent GC cohorts. Correlations of the EMD score with tumor mutation burden, tumor purity, aneuploidy score, tumorigenic pathways, TME characteristics, and FTO/HDAC1 ratio were measured. *In vitro* experiments were performed to demonstrate the correlation between FTO and the epithelial–mesenchymal transition pathway, which suggested FTO as a targetable vulnerability for GC patients with a high EMD score. Altogether, by comprehensively analyzing the epigenetic modification patterns of 1518 GC patients, we identified a novel stromal-activated subtype with poor survival and resistance to immunotherapy, which might benefit from the combined immune checkpoint inhibition therapy with FTO inhibition.

## 1 Introduction

Gastric cancer (GC), the fifth most common cancer and the third most common cause of cancer deaths in the world, remains a non-negligible health problem and social burden globally (Smyth et al., 2020). Patients with early-stage GC featured a 5-year overall survival (OS) rate of more than 60%, for whom surgical resection is the best option (Thrift and El-Serag, 2020). For patients with advanced GC, chemotherapy represented by fluoropyrimidines and platinum significantly improves survival and the quality of life. Unfortunately, due to the high frequency of advanced stage at diagnosis and chemotherapy resistance, the 5-year overall survival rate of advanced GC is still less than 5% (Yuan et al., 2020). As a complex disease with molecular and clinical heterogeneity, GC shows versatile phenotypes in initiation, progression, and even the response to treatment among different patients. Thus, more effective individualized strategies both in diagnosis and therapy for GC are urgently needed to be explored.

Epigenetic modification, mainly including DNA methylation, histone modification, and RNA modification, is a significant regulatory mechanism of diverse physiological or pathological processes (Zhao et al., 2021a). Histone modification mainly refers to the post-translational modifications (PTMs) that occur in the N-terminal tails of histone proteins of nucleosomes, including but not limited to methylation, acetylation, and ubiquitination (Zhao and Shilatifard, 2019). Generally, histone modifications (such as H3K4me1, H3K4me3, and H3K27ac) enriched at the enhancer or promoter region presumably facilitate the transcription process of the targeted genes (Bannister and Kouzarides, 2011). N6-methyladenosine (m6A), defined as methylation of adenosine at the N6 position, is one of the most abundant RNA modification types in eukaryotic species including mammals, plants, insects, yeast, and certain viruses (Gu et al., 2020). Recently, accumulating evidence has suggested the extensive interactions between histone and m6A modifications, which trigger epigenetic remodeling and cause profound impacts on various aspects of cancer progression, including the resistance to medical treatment. (Huang et al., 2019; Li et al., 2020a; Yang et al., 2021a; Li et al., 2021). For example, Li et al. uncovered a SOX4/EZH2/METTL3 axis in TMZ-resistant glioblastoma (GBM), in which EZH2 regulates the METTL3 expression *via* an H3K27me3 modification-independent manner, and METTL3 leads to nonsense-mediated mRNA decay of EZH2 reversely (Li et al., 2021). Moreover, Li et al. found that the m6A reader YTHDC1 physically interacts with and recruits KDM3B to m6A-associated chromatin regions, promoting H3K9me2 demethylation and gene expression, establishing a direct link between m6A and histone modification (Li et al., 2020a). Similar interactions have also been observed in GC. Yang et al.’s study suggested that HDAC3 regulates the FTO (fat-mass and obesity-associated protein) expression in a FOXA2-dependent manner, thus promoting the proliferation, migration, and invasion of GC cells (Yang et al., 2021a).

The tumor microenvironment (TME), known as the soil of the tumor seed, which plays a pivotal role in tumorigenesis and anti-tumor immunity, has been recently reported to be shaped by various epigenetic modifications (Huang et al., 2012; Gu et al., 2021). For instance, Yin et al. reported that EZH2 depletion increased generation of the IL-15 receptor (IL-15R), CD122 (+) NK precursors, and mature NK progeny from both mouse and human hematopoietic stem and progenitor cells, demonstrating the impact of histone modification H3K27me3 on early NK cell differentiation (Yin et al., 2015). Wang et al. found that METTL3/14-deficient tumors increased cytotoxic tumor-infiltrating CD8^+^ T cells and elevated secretion of IFN-γ, CXCL9, and CXCL10 in the TME *in vivo*, and inhibition of METTL3/14 enhanced response to anti-PD-1 treatment in pMMR-MSI-L CRC and melanoma (Wang et al., 2020). However, the epigenetic modification patterns and their association with TME, prognosis, and therapeutic response in GC remain poorly investigated.

In this study, we aimed to characterize the extensive crosstalk between histone and m6A modifications in GC, trying to explain the molecular and clinical heterogeneity of GC from the perspective of epigenetic dysregulations.

## 2 Materials and Methods

### 2.1 Data Acquisition and Processing

**FIGURE 1:**
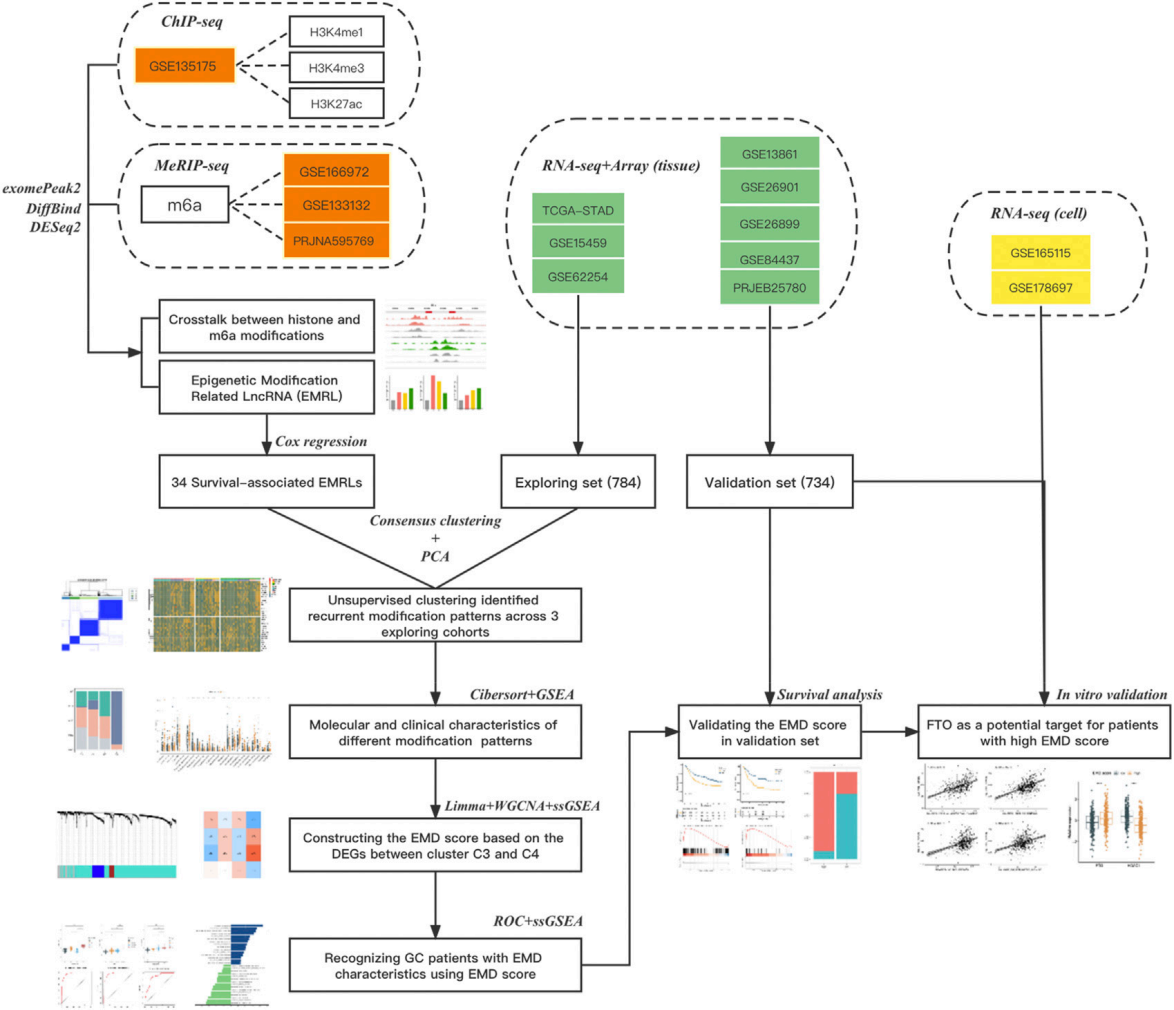
Flow chart of this study. Three types of data from 14 datasets were used in this study: datasets in red refer to the epigenetic data (ChIPseq or MeRIPseq) targeting on histone or m6A modifications, datasets in green refer to the RNAseq or array data with complete clinical information (survival or ICI response), and datasets in yellow refer to the RNAseq data of cell lines with or without FTO depletion. The main method used in each step was also listed.

As shown in the flowchart of this study (Figure 1), we obtained ChIP-seq data of three histone modification types (H3K4me1, H3K4me3, and H3K27ac) in four gastric cell lines (GES-1, SNU719, NCC24, and YCC10), and MeRIP-seq data of m6A in three GC cell lines (AGS, BGC823, and SGC7901) from previously published literature reports with the access number of GSE135175 (Okabe et al., 2020), GSE166972 (Chen et al., 2021a), GSE133132 (Yue et al., 2019), and PRJNA595769 in the Gene Expression Omnibus (or the European Nucleotide Archive), respectively (Supplementary Table S1). For ChIP-seq, raw sequencing reads were aligned using Hisat2 (Kim et al., 2019) with default parameters to the hg19. Sambamba (Tarasov et al., 2015) was used to remove PCR duplicates and obtain the uniquely mapped reads. For MeRIP-seq, raw sequencing reads were aligned using Hisat2 with default parameters to the hg38. Samtools (Li et al., 2009) was used to remove the reads with a mapping quality below 30.

Totally 1518 GC patients from eight independent cohorts with complete transcriptomic data and clinical information were obtained from The Cancer Genome Atlas TCGA-STAD (stomach adenocarcinoma, https://portal.gdc.cancer.gov), Gene Expression Omnibus [GSE62254 (Cristescu et al., 2015), GSE15459 (Ooi et al., 2009), GSE13861 (Cho et al., 2011), GSE26899 (Oh et al., 2018), GSE26901 (Oh et al., 2018), GSE84437, https://www.ncbi.nlm.nih.gov/gds], and European Nucleotide Archive (PRJEB25780, https://www.ebi.ac.uk) (Supplementary Table S1). Cohorts were divided into exploring set (TCGA-STAD, GSE15459, and GSE62254) and validation set (GSE13861, GSE26899, GSE26901, GSE84437, and PRJEB25780) (Figure 1). Patients with prior or synchronous malignancy diagnoses or patients who survived less than 30 days were excluded from this study. Sva (Jeffrey, 2020) was used to correct the non-biological batch effects among different cohorts.

### 2.2 Collection of Epigenetic Regulators

**FIGURE 2:**
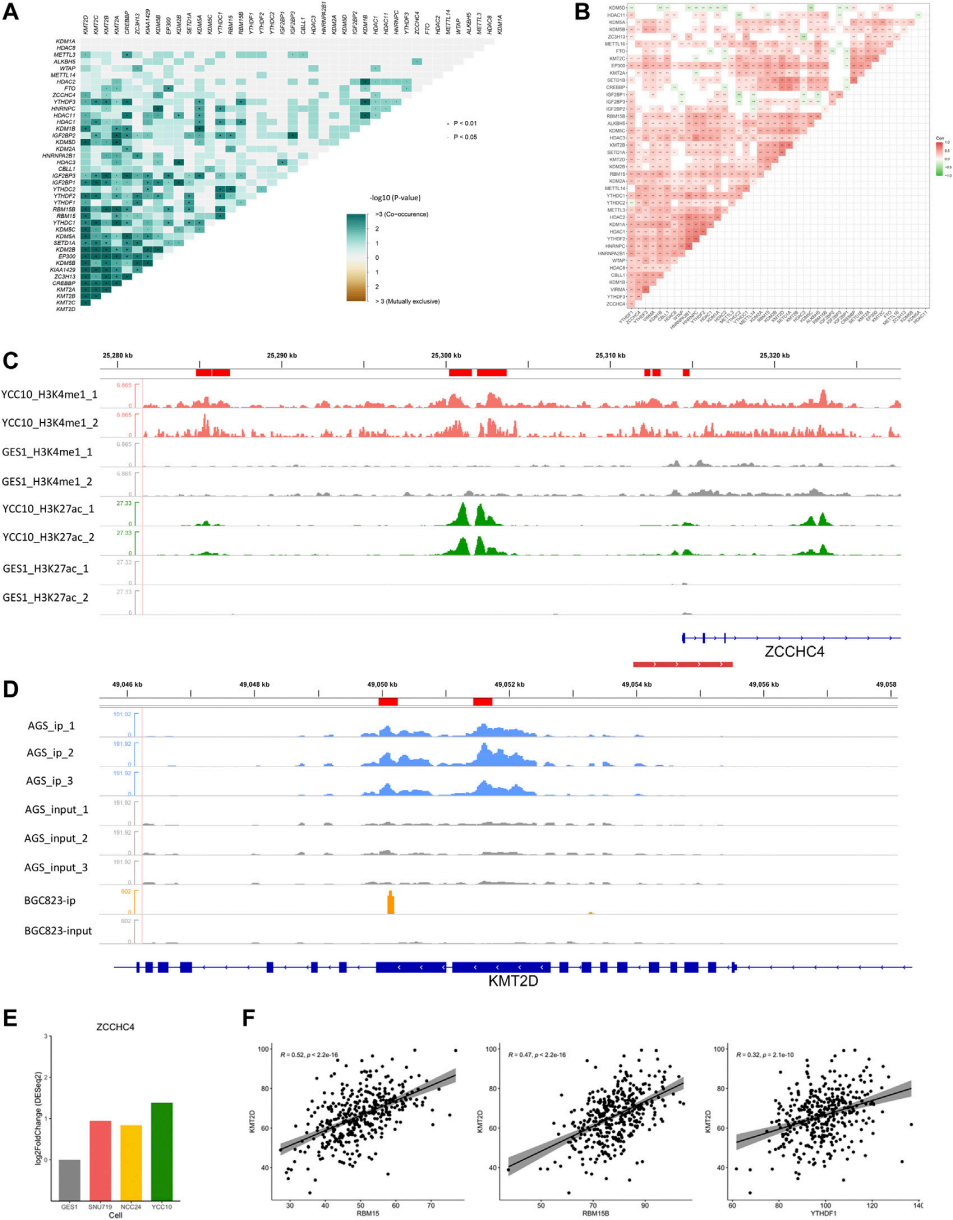
Crosstalk between regulators of histone and m6A modifications. **(A)** Co-mutation heatmap of epigenetic regulators in the TCGA-STAD cohort. **(B)** Co-expression heatmap of epigenetic regulators in the TCGA-STAD cohort. **(C)** Differential H3K4me1 and H3K27ac modifications in the m6A regulator ZCCHC4. Peaks in red and green refer to the H3K4me1 and H3K27ac modifications in GC cell lines, respectively, and gray refers to the corresponding modification in GES-1 as control. The red line at the bottom refers to the promoter region. **(D)** Differential m6A modification in the histone modification regulator KMT2D. Peaks in blue and orange refer to the immunoprecipitation (IP) signals of AGS and BGC823 cell lines, respectively. Peaks in gray refer to the corresponding input signals. **(E)** Differential expression of ZCCHC4 between GC cell lines and GES-1 (*p* < 0.05). **(F)** Correlations of KMT2D with the m6A regulators including RBM15, RBM15B, and YTHDF1 (Spearman, *p* < 0.05).

Epigenetic regulators, defined as the key genes that play crucial roles in the regulation or function of epigenetic modifications, mainly include the methyltransferases/demethylases of histone methylation (H3K4me1 and K3K4me3), acetylases/deacetylases of histone acetylation (H3K27ac), and writers/readers/erasers of m6A modification. In our study, totally 43 epigenetic regulators (Supplementary Table S1), including histone methyltransferases (SETD1A, SETD1B, KMT2A, KMT2B, KMT2C, and KMT2D), histone demethylases (KDM1A, KDM1B, KDM2A, KDM2B, KDM5A, KDM5B, KDM5C, and KDM5D), histone acetylases (CREBBP and EP300), histone deacetylases (HDAC1, HDAC2, HDAC3, HDAC8, and HDAC11), m6A writers (METTL14, METTL3, METTL16, RBM15, RBM15B, ZC3H13, ZCCHC4, WTAP, CBLL1, and VIRMA), m6A readers (YTHDC1, YTHDC2, YTHDF1, YTHDF2, YTHDF3, IGF2BP1, IGF2BP2, IGF2BP3, HNRNPA2B1, and HNRNPC), and m6A erasers (ALKBH5 and FTO), were collected from the published literature (Zhao and Shilatifard, 2019; Zhao et al., 2021a; Deng et al., 2021) for our study. All the included regulators have been experimentally demonstrated to regulate one or more modification types, most of which were reported to participate in tumorigenesis or progression of GC (Yue et al., 2019; Wu et al., 2021a). Maftools (Mayakonda et al., 2018) was used to identify the mutation (Figure 6F) or co-mutation (Figure 2A) events of epigenetic regulators in the TCGA-STAD cohort. Co-occurrence events with *p* values less than 0.05 were defined as the co-mutation events (Supplementary Table S2).

### 2.3 Identification of the Epigenetic Modification-Related Gene

For histone modifications (H3K4me1, H3K4me3, and H3K27ac), DiffBind (Stark and Brown, 2011) was used to identify the differentially-histone-modfied site (DHMS) between GC cell lines and GES-1 according to the criteria of |fold-change| >1 and *p* < 0.05. BigWig data were downloaded from the GEO with the access number GSE135175. DESeq2 (Love et al., 2014) was used to identify the differentially expressed gene (DEG) between GC cell lines and GES-1 with the criteria of |log_2_FC| >1 and *p* < 0.05. For m6A modification, exomePeak2 (Wei, 2020) was used to identify the m6A-modified site (MMS) in each GC cell line, according to the criteria of |fold-change|>1 and *p* < 0.05. The Wilcoxon test was used to identify the correlatively expressed gene (CEG) of the 22 m6A regulators using the criteria of |r|>0.3 and adjusted *p* < 0.05.

Generally, histone methylations/acetylations (such as H3K4me1, H3K4me3, and H3K27ac) enriched at enhancers or promoters, as well as m6A modifications enriched at coding sequence (CDS) or the 3′ untranslated region (3′ UTR) region, presumably facilitate the expression of target genes (Bannister and Kouzarides, 2011; Shi et al., 2019). Thus, those genes which satisfy one of the following criteria were defined as the epigenetic-modification-related gene (EMRG):

1) At least one DHMS was located in the promoter of the DEG, and the expression of the DEG changed in the same direction as the DHMS in at least one GC cell line (SNU719, NCC24, and YCC10);
2) At least one MMS was located in the CEG in at least one GC cell line (AGS, BGC823, and SGC7901), and the CEG positively correlated (r > 0.3, Wilcoxon test) with at least one of the m6A writers/readers, or negatively correlated (r < −0.3, Wilcoxon test) with at least one of the m6A erasers.

The promoter region was defined as 3 kb upstream and downstream of the transcriptional start site (TSS) in each gene. The correlations between the CEG and m6A regulators were calculated in the TCGA-STAD cohort. Long non-coding RNAs (LncRNAs) of the epigenetic modification-related Gene were defined as the epigenetic-modification-related LncRNA (EMRL), according to the GENCODE annotations (Yang et al., 2021b).

### 2.4 Unsupervised Clustering in the Exploring Set

By performing univariate Cox hazard analysis in the TCGA-STAD cohort, we selected 34 survival-associated EMRLs (*p* < 0.01) for further study. Based on the 34 survival-associated EMRLs, ConsensusClusterPlus (Wilkerson and Hayes, 2010) was applied to perform unsupervised clustering in the exploring set with the following parameters: maxK = 7, cluster algorithm = km, and correlation method = Euclidean.

### 2.5 Construction of the EMD Score

We first identified 1,674 DEGs between the epigenetic-modification-dysregulated (EMD) subtype (C4) and cluster C3 using limma (Ritchie et al., 2015). (Supplementary Table S7) Weighted gene co-expression network analysis (WGCNA) (Langfelder and Horvath, 2008) was then performed based on the DEGs to recognize the most positively correlated (Meturquois, Spearman-r = 0.75) and negatively correlated (MEblue, Spearman-r = −0.61) gene modules relating to cluster C4 (Figures 6A–C). We further chose 147 (set1) and 64 (set2) genes from the turquoise and blue modules, respectively, according to their correlation coefficients with the EMD subtype and corresponding modules (Figure 6D; Supplementary Table S8). The single sample gene set enrichment analysis (ssGSEA) algorithm in R package GSVA (Hänzelmann et al., 2013) was used to estimate the relative abundance (ssGSEA value) of set1 and set2, respectively, for each patient in the GC cohorts. The EMD score was defined as the ratio of the two ssGSEA values. Log transition of the ratio was also performed to make the score less discrete. For each GC patient, the EMD score was calculated as follows:

$$EMD\;score=\log_{2}\left\{\;ssGSEA\;score\;\left(set1\right)/\;ssGSEA\;score\;\left(set2\right)\right\}.$$

### 2.6 TME Characterization and Function Enrichment Analysis

CIBERSORT (Chen et al., 2018) and ESTIMATE (Yoshihara et al., 2013) were used to evaluate the relative abundance of immune infiltration and stromal components in tumor samples, respectively. Deconvolution results of CIBERSORT were evaluated by a derived *p*-value (*p* < 0.05) to filter out the samples with less significant accuracy. The tumor immune dysfunction and exclusion (TIDE) algorithm (Jiang et al., 2018) was used to measure the antitumor immunity features of each patient. ClusterProfiler (Yu et al., 2012) was used to perform gene set enrichment analysis (GSEA), using the hallmark gene sets downloaded from the molecular signatures database (MSigDB).

### 2.7 Cell Culture and Treatment

Human GC cell lines (SNU719 and SGC7901) were purchased from American Type Culture Collection (Manassas, United States). SNU719 and SGC7901 cells were cultured in RPMI-1640 medium (Gibco) with 10% fetal bovine serum (Gibco). Cells were maintained at 37°C and 5% CO_2_. SNU719 and SGC7901 cells were plated onto 6-well plates and reached 70–80% cell confluence on the day of treatment. Cells were divided into the BS group and the control group. The BS group was treated with the FTO inhibitor (Brequinar sodium, V17016, 5 umol/l, InvivoChem), and the DMSO group was treated with an equivalent DMSO concentration as control for 48 h.

### 2.8 Western Blot

48 h after treatment, the EMT markers in SNU719 and SGC7901 were analyzed by Western blot. In brief, whole protein samples of all groups were extracted, and the concentration was detected by using the Pierce BCA Protein Assay Kit (Thermo 23225). Equal amounts of protein (20 ug per well) were loaded, and the samples were separated by 10% sodium dodecyl sulfate–polyacrylamide gel electrophoresis. Then, the proteins were transferred to 0.45 μm polyvinylidene fluoride (PVDF) membranes. The membranes were blocked with 5% nonfat milk and incubated with primary antibodies at 4°C overnight. Later, the membranes were washed with TBST three times and incubated with the secondary HRP antibody at room temperature for 2 h. The primary antibodies used in this research were as follows: E-cadherin (20874-1-AP, 1:2000, Proteintech), N-cadherin (22018-1-AP, 1:2000, Proteintech), Vimentin (10366-1-AP, 1:5,000, Proteintech), and TWIST1 (25465-1-AP, 1:1,000, Proteintech). β-actin (3700T, 1:5,000, CST) was used as a loading control.

### 2.9 Statistical Analysis

All statistical analyses were performed using R (v4.0.2) (Team, R. C 2020) and its appropriate packages. *p*-values <0.05 were regarded as statistically significant.

## 3 Results

### 3.1 Crosstalk between Regulators of Histone and m6A Modifications

First, we examined the interactions of the four modification types in the TCGA-STAD cohort. Totally, 211 co-mutation events (*p* < 0.05) and 266 co-expression events (|r|>0.3, *p* < 0.05) were observed in the 43 epigenetic regulators (Figures 2A, B; Supplementary Table S2). These correlations were further illustrated by the ChIP/MeRIP-seq data from GC cell lines. Specifically, m6A regulators including ZCCHC4, IGF2BP3, and VIRMA were differentially expressed between the GC cell lines and GES-1, which were accompanied by differential histone modifications in their TSS regions (Figure 2; Supplementary Figure S1). Similarly, the histone modification regulators, SETD1A and KMT2D, which showed positive correlations with the m6A regulators (RBM15, RMM15B, and YTHDF1), had differential m6A modification in their exons (Figure 2; Supplementary Figure S1; Supplementary Table S2). These findings revealed the active crosstalk between histone and m6A modifications, both in genome and transcriptome levels.

### 3.2 Identification of the EMRG and EMRL

**FIGURE 3:**
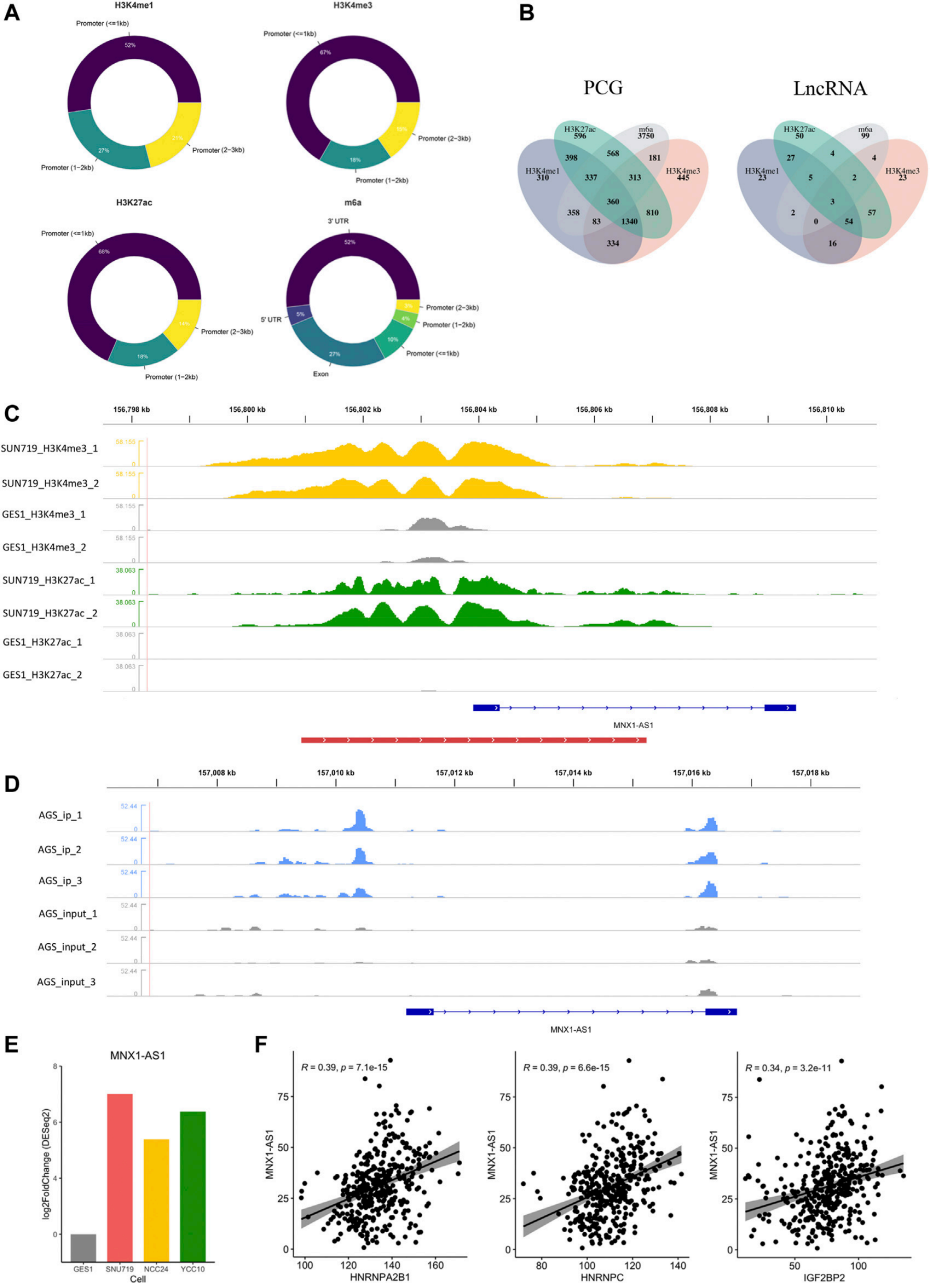
Identification of epigenetic modification-related LncRNA (EMRL). **(A)** Doughnut charts showed the distribution of DHMS or MMS in each modification type. **(B)**Venn diagram showed the overlaps of EMRG (or EMRL) regulated by different modification types. PCG refers to the protein-coding genes, and LncRNA refers to the long non-coding RNAs. **(C)** MNX1-AS1 was potentially regulated by H3K4me3 and H3K27ac in GC cell lines. Peaks in yellow and green refer to H3K4me3 and H3K27ac, respectively, while gray ones refer to the corresponding modification in GES-1 as control. The red line at the bottom refers to the promoter region. **(D)** m6A modification regions of MNX1-AS1 in the AGS cell line. Peaks in blue refer to the immunoprecipitation (IP) signals. Peaks in gray refer to the input signals. **(E)** Differential expression of MNX1-AS1 in GC cell lines and GES-1 (*p* < 0.05). **(F)** Correlations of MNX1-AS1 with the m6A regulators including HNRNPC, HNRNPA2B1, and IGF2BP2 (Spearman, *p* < 0.05).

To generally characterize the transcriptome landscape altered by the four epigenetic modification types, we then identified the dysregulated genes associated with each of the modification types. Using the criteria described earlier, we finally identified 4,999 EMRGs of H3K4me1, 3,693 EMRGs of H3K4me3, 4,095 EMRGs of H3K27ac, and 6,117 EMRGs of m6A. Previous studies suggested that some histone modifications (such as H3K4me1, H3K4me3, and H3K27ac) enriched at the enhancer or promoter region presumably facilitate the transcription of targeted genes (Bannister and Kouzarides, 2011). Similarly, unbalanced distribution was also observed in the m6A modification, with more than 40% of all modification sites in mRNA being present in 3′ UTRs) (Ke et al., 2015; Kan et al., 2022). Consistent with the previous studies, most of MMSs were located at the 3′UTR, followed by exons, and then the promoter region (Figure 3A). Meanwhile, for the three histone modification types, most of DHMSs were located within the promoter (<1 kb), followed by the promoter (1–2 kb) and promoter (2–3 kb) (Figure 3A). Among the EMRGs, nearly 50% of genes were simultaneously regulated by more than one modification type, which was another evidence for the crosstalk between different modification types. Specifically, about 360 protein-coding genes (PCGs) were co-regulated by the four modification types (Figure 3B).

As another key member of epigenetic regulation, LncRNA was demonstrated to participate in various tumorigenic processes due to their extensive biological functions in the transcriptome level (Sempere et al., 2021; Kan et al., 2021). To further characterize the epigenetic modification patterns with clinical relevance in GC, we also identified 370 EMRLs (Supplementary Table S3) from the EMRG. Interestingly, three EMRLs (PAXIP1-AS2, MNX1-AS1, and PVT1) were co-regulated by the four modification types (Figure 3B). LINC00511, previously reported as an oncogene in several solid tumors (Wu et al., 2020; Peng et al., 2021; Dong et al., 2021), was significantly modified with H3K4me1 (Supplementary Figure S2) and simultaneously overexpressed in the three GC cell lines (Figure 3F). Similarly, concomitant histone modification and overexpression were observed in LINC01091 (Supplementary Figure S2). Moreover, we found MNX1-AS1, another oncogene in multiple cancers (Wu et al., 2019; Li et al., 2020b; Li et al., 2020c; Wu et al., 2021b), was simultaneously regulated by both histone and m6A modifications, with differential expression across three GC cell lines and positive correlations to multiple m6A regulators (Figures 3C–F). The aforementioned findings shed light on the close connections inside epigenetic regulators including epigenetic modifications and LncRNA, offering a new perspective for exploring the complicated regulation network of the epigenome.

### 3.3 Unsupervised Clustering Based on EMRL Identified an EMD Subtype of GC

**FIGURE 4:**
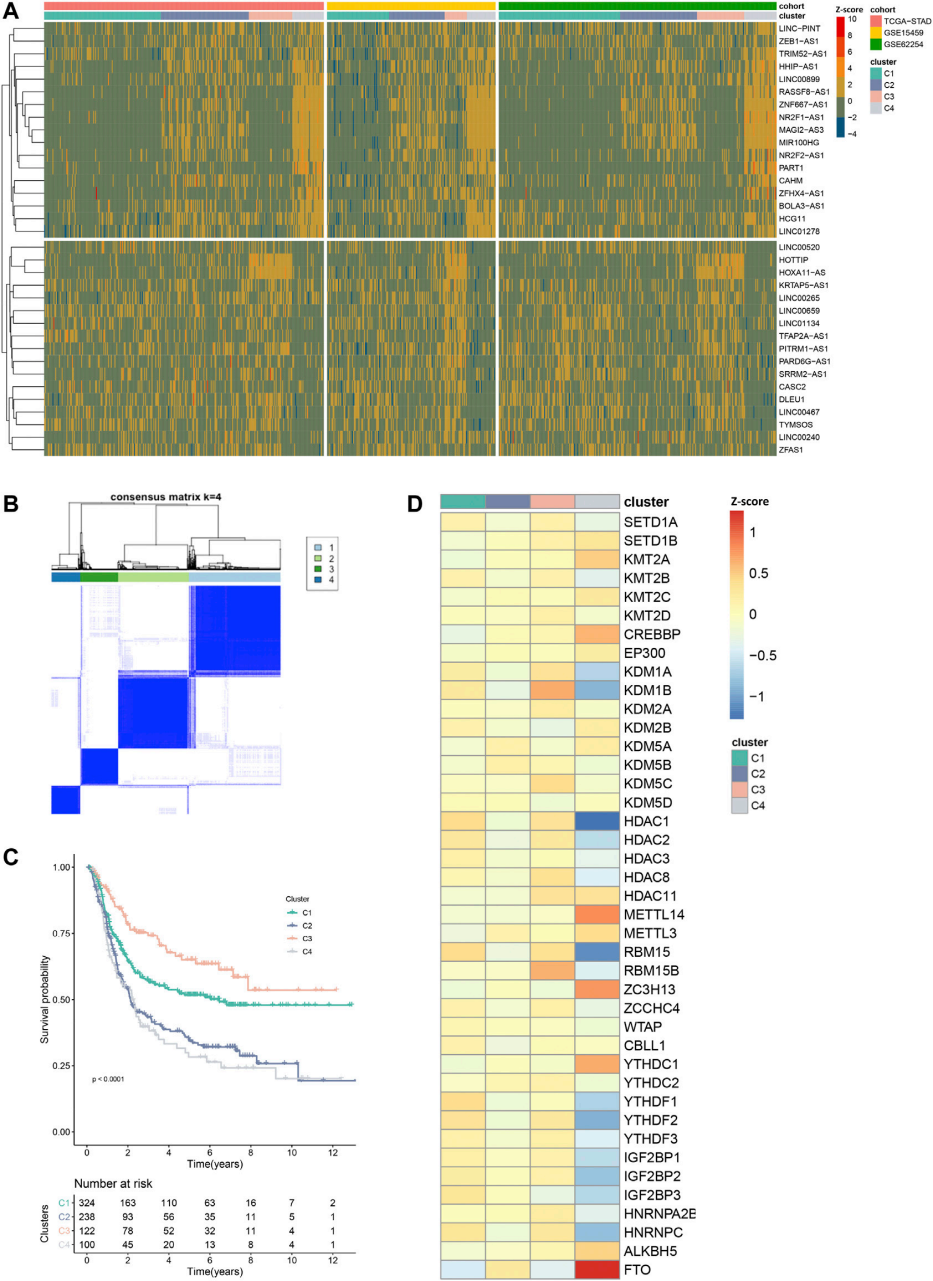
Distinctive epigenetic modification patterns across three GC cohorts recognized by EMRL. **(A)** Heatmap showed the highly concordant expression patterns of the three GC cohorts in the exploring set. **(B)** Consensus unsupervised clustering divided the exploring set into four distinct clusters. **(C)** Kaplan–Meier curves showed the different overall survival of the four clusters. **(D)** Heatmap showed the distinct expression patterns of the epigenetic regulators in the exploring set. VIRMA and METTL16 were excluded in this section due to lack of expression information in the GSE15459 or GSE62254 cohort.

Next, we set out to characterize the modification patterns in the exploring set. Through univariate Cox regression analysis, we selected 34 EMRLs with a significant prognosis value (*p* < 0.01) for further study (Supplementary Table S4). Unsupervised clustering was then performed based on the 34 survival-associated EMRLs, which divided the exploring set into four distinct clusters (Figures 4A, B; Supplementary Figure S3A; Supplementary Table S5). Surprisingly, extremely similar expression patterns were observed across the three independent GC cohorts (TCGA-STAD, GSE15459, and GSE62254), especially in cluster C4. Specifically, the C4 population of each cohort was characterized by strikingly elevated EMRLs including ZEB1-AS1, NR2F1-AS1, MIR100HG, ZFHX4-AS1, and PART1 and the suppression of EMRLs including HOTTIP, HOXA11-AS, CASC2, and LINC00467. Moreover, a significant difference in prognosis was observed among the four clusters, with cluster C4 having the worst survival and cluster C3 the best (Figure 4C).

Consistently, unbalanced modification patterns both of histone and m6A modifications were observed in cluster C4. Specifically, cluster C4 was characterized by the high expression of multiple histone modification writers (SETD1B, KMT2A, and CREBBP) and the low expression of histone modification erasers (KDM1A, KDM1B, HDAC1, and HDAC2). While in the m6A modification, high-expressed erasers (FTO and ALKBH5) and low-expressed readers (YTHDF2, YTHDF3, IGF2BP2, IGF2BP3, and HNRNPC) were observed in cluster C4 (Figure 4D). Then, we further explored the mutation profiling of cluster C4. Interestingly, cluster C4 was also featured with a distinctive mutation pattern, with rarely detected mutations of the epigenetic regulators (Supplementary Figure S3B). Given the distinctive modification patterns in transcriptome and genome levels and the poor prognosis of cluster C4, we named it the epigenetic-modification-dysregulated (EMD) subtype in further study. In this section, using survival-associated EMRLs, we identified a conserved EMD subtype with distinctive modification patterns in GC.

### 3.4 Molecular and Clinical Characterization of the EMD Subtype

**FIGURE 5:**
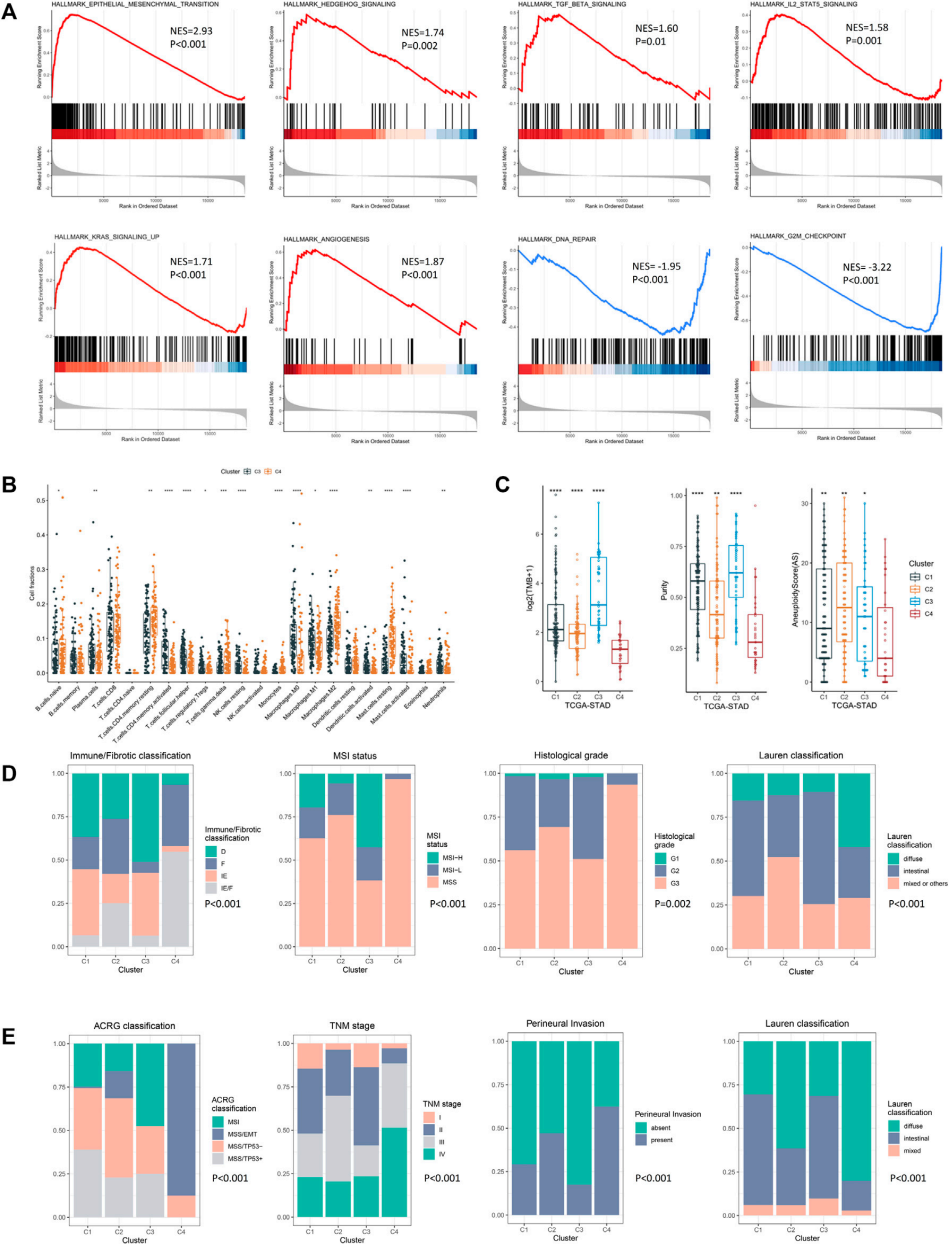
Molecular and clinical characteristics of four epigenetic modification patterns. **(A)** GSEA analysis identified the differentially activated pathways of cluster C4 compared with cluster C3. **(B)** CIBERSORT analysis estimated the relative ratio of tumor-infiltrating cells in clusters C3 and C4. Each value was defined as the relative ratio compared to the 22 human hematopoietic cell types contained in CIBERSORT. **(C)** Different TMB, tumor purity, and aneuploidy score among four clusters in the TCGA-STAD cohort. **(D)** Clinical relevance of the EMD subtype in the TCGA-STAD cohort. **(E)** Clinical relevance of the EMD subtype in the GSE62254 cohort (*p < 0.05, **p < 0.01, ***p < 0.001, and ****p < 0.0001).

To figure out the mechanism underlying the poor prognosis of the EMD subtype, we comprehensively explored the molecular and clinical characteristics of the EMD subtype by comparing it with cluster C3 (cluster with the best prognosis) in the exploring set. In the GSEA analysis, multiple tumorigenic pathways including the epithelial–mesenchymal transition (EMT, *p* < 0.001), Hedgehog (*p* = 0.002), TGF-β (*p* = 0.01), IL2-STAT5 (*p* = 0.001), KRAS (*p* < 0.001), and angiogenesis (*p* < 0.001) were significantly activated in the EMD subtype (Figure 5A), whereas pathways including DNA repair (*p* < 0.001) and G2M checkpoint (*p* < 0.001) were observed to be significantly suppressed in the EMD subtype (Figure 5A).

Then, we performed CIBERSORT analysis to explore the TME characteristic of the EMD subtype. Compared with cluster C3, the EMD subtype showed a significantly higher ratio of macrophages M2 (*p* < 0.001) and resting CD4^+^ T cell (*p* < 0.01) and a significantly lower ratio of macrophages M1 (*p* < 0.05) and activated CD4^+^ T cell (*p* < 0.001) (Figure 5B). Moreover, a relatively lower CD4^+^/CD8^+^ T cell ratio was observed in the EMD subtype (*p* < 0.05, Supplementary Figure S4A), which usually indicates more advanced tumors or immune deficiency in cancer patients. In the TCGA-STAD cohort, we also observed a significant decrease of tumor mutation burden (TMB, *p* < 0.001), tumor purity (*p* < 0.001), and aneuploidy score (AS, *p* < 0.05) in the EMD subtype (Figure 5C).

In addition to the distinctive molecular characteristics, the EMD subtype also showed extensive correlations to various clinical characteristics of GC (Supplementary Table S6). In the TCGA-STAD cohort (Figure 5D), we found the EMD subtype as a good indicator for the IE/F subtype of the newly reported immune/fibrotic classification (Bagaev et al., 2021) (*p* < 0.001). Besides, more patients with MSS subtype (*p* < 0.001), poor differentiation (*p* = 0.002), or diffuse histotype (*p* < 0.001) were observed in the EMD subtype. While in the GSE62254 cohort (Figure 5E), the EMD subtype mostly overlapped with the MSS/EMT subtype of ACRG classification (Cristescu et al., 2015) (*p* < 0.001). Similarly, more patients with advanced TNM stage (*p* < 0.001), perineural invasion (*p* < 0.001), or diffuse histotype (*p* < 0.001) were observed in the EMD subtype.

These findings suggested distinctive stromal-activated and immune-suppressed characteristics of the EMD subtype, which may account for the poor prognosis of this subtype.

### 3.5 Construction of the EMD Score in the Exploring Set

**FIGURE 6:**
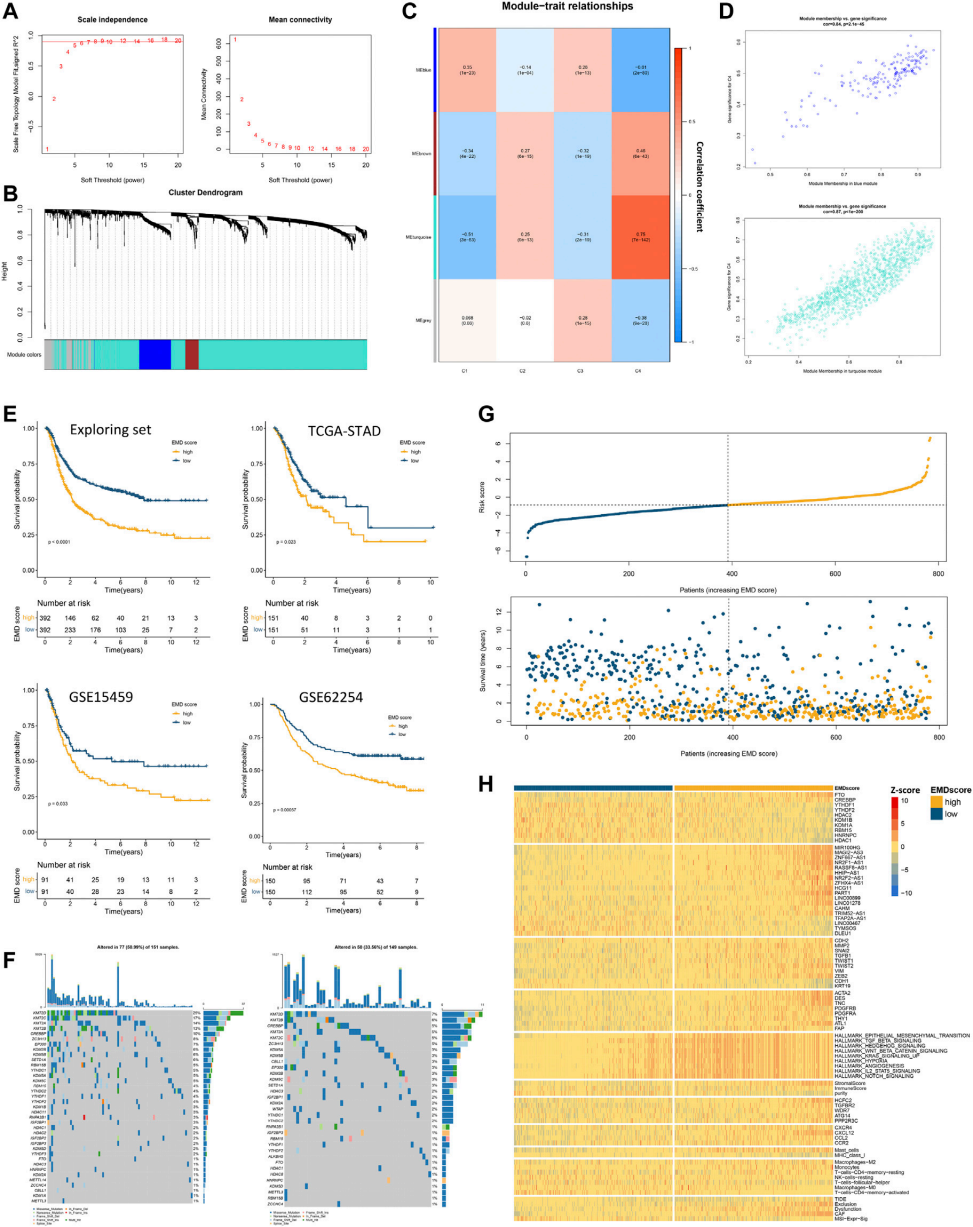
Construction of the EMD score in the exploring set. **(A–D)** WGCNA analysis based on 1674 DEGs in the exploring set. **(E)** Kaplan–Meier curves showed the different overall survival between the high- and low-EMD score groups in the exploring set. Patients were separated into the high and low groups according to the median EMD score of each cohort. **(F)** Mutation landscape of epigenetic regulators in high- and low-EMD score groups in the TCGA-STAD cohort. **(G)** Risk curves showed more frequent death events with the increasing EMD score in the exploring set. Dots in yellow referred to death events observed in the cohorts. **(H)** Heatmap showed the close correlations of the EMD score with various molecular characteristics including epigenetic regulators, EMRLs, TME markers, oncogenes, and tumorigenic pathways. All the values were scaled using the Z-score.

Since clusters C3 and C4 showed marked differences in prognosis and molecular characteristics, we chose clusters C3 and C4 for further study. To expand the applicability of the EMD subtype, an EMD score was then constructed based on the DEGs between the EMD subtype (C4) and cluster C3 using WGCNA (Figures 6A–D) and ssGSEA algorithms. Strikingly, the EMD score was closely related to the prognosis in the exploring set, where the increasing EMD score was accompanied by more death events (Figure 6G) and worse survival (Figure 6E).

Consistent with the EMD subtype, extensive correlations of the EMD score with stromal-activated and immune-suppressed TME characteristics could be observed in the exploring set (Figure 6H; Supplementary Tables S9, S10). Specifically, patients with a high EMD score showed activation of multiple pathways (EMT, TGF-β, and Hedgehog), elevated stromal scores, high abundance of cancer-associated fibroblast (CAF) and mast cells, whereas those with a low EMD score showed low MHC-I expression, low infiltration of activated CD4^+^ T cells, and low M1/M2 ratio. These were further evidenced by upregulated markers of EMT activation (ZEB2, TWIST1, SNAI2, MMP2, CDH2, VIM, etc.), CAF (ACAT2, DES, TNC, PDGFRB, etc.), and immune-suppressed chemokine axis (CXCR4, CXCL12, CCL2, and CCR2) with the increasing EMD score. Moreover, we also observed the overexpression of multiple immune evasion-related genes (CREBBP, HCFC2, TGFBR2, WDR7, ATG14, and PPP2R3C), which were experimentally demonstrated (Lawson et al., 2020), in the patients with a high EMD score. Additionally, in the TIDE analysis, we found the EMD score significantly correlated with the exclusion, dysfunction, CAF, and MSI signature, which implied the potential of the EMD score in predicting the ICI efficacy. Similar to the EMD subtype, a high EMD score indicated a lower frequency of regulator mutation (34%, 51%, Figure 6F). These findings suggested the EMD score as a good indicator for recognizing the EMD subtype in GC.

### 3.6 Recognizing Patients with EMD Characteristics in the Validation Set Using the EMD Score

**FIGURE 7:**
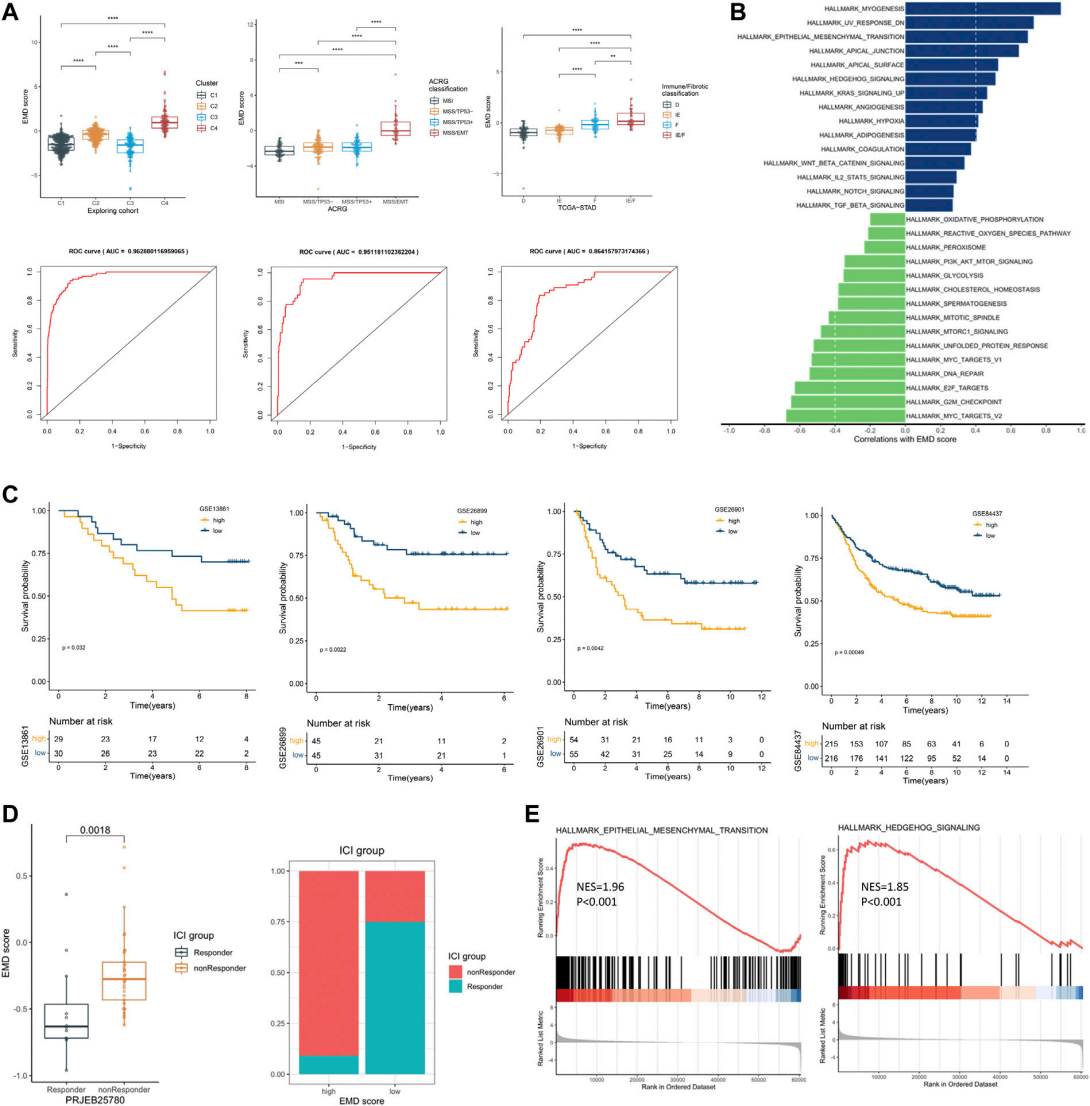
Recognizing patients with EMD characteristics using the EMD score. **(A)** Differential EMD score of subtypes in three classification proposals. The ROC below showed the performance of the EMD score in recognizing the EMD, MSS/EMT, and IE/F subtype in the corresponding cohort. **(B)** Hallmark pathways correlated with the EMD score in the validation set. **(C)** Kaplan–Meier curves showed the different overall survival between the high and low EMD groups in the validation set. Patients were separated into high and low groups according to the median EMD score of each cohort. **(D)** Correlations of the EMD score with the ICI outcome in the cohort PRJEB25780. Patients were divided into high or low groups according to the lower quartile (Q1) of the EMD scores (right panel). **(E)** GSEA analysis suggested significant activated EMT and Hedgehog pathways in non-responders compared with the responder.

We first tested the correlations of the EMD score with the four clusters we identified earlier, ACRG classification, and immune/fibrotic classification. Strikingly, the EMD score showed an excellent performance in recognizing the EMD subtype (AUC = 0.96, Figure 7A) in the exploring set, as well as the MMS/EMT subtype (AUC = 0.95, Figure 7A) of ACRG classification and the IE/F subtype of the immune/fibrotic classification (AUC = 0.86, Figure 7A).

To further validate the ability of the EMD score in predicting prognosis and response to ICI treatment, we calculated the EMD score for patients in four independent GC cohorts. As we expected, a high EMD score was closely related to a worse OS (*p* < 0.05, Figure 7C) in each cohort of the validation set. Strong correlations between the EMD score and multiple pathways (EMT, TGF-β, Hedgehog, etc. Figure 7B; Supplementary Table S11) could also be observed in the validation set. In the cohort PRJEB25780, we also observed a distinct difference in the response rate to immune checkpoint inhibitor (ICI) treatment between high and low EMD score groups (10 *vs.* 75%, *p* = 0.0018, Figure 7D). Moreover, non-responders were featured with significant activated EMT (NES = 1.96, *p* < 0.001) and Hedgehog (NES = 1.85, *p* < 0.001) pathways compared with the responders (Figure 7E). These findings successfully validated the ability of the EMD score in recognizing the patients with EMD characteristics and the potential of recognizing patients sensitive to ICI treatment in GC.

### 3.7 FTO as a Potential Target for Patients with High EMD Score

**FIGURE 8:**
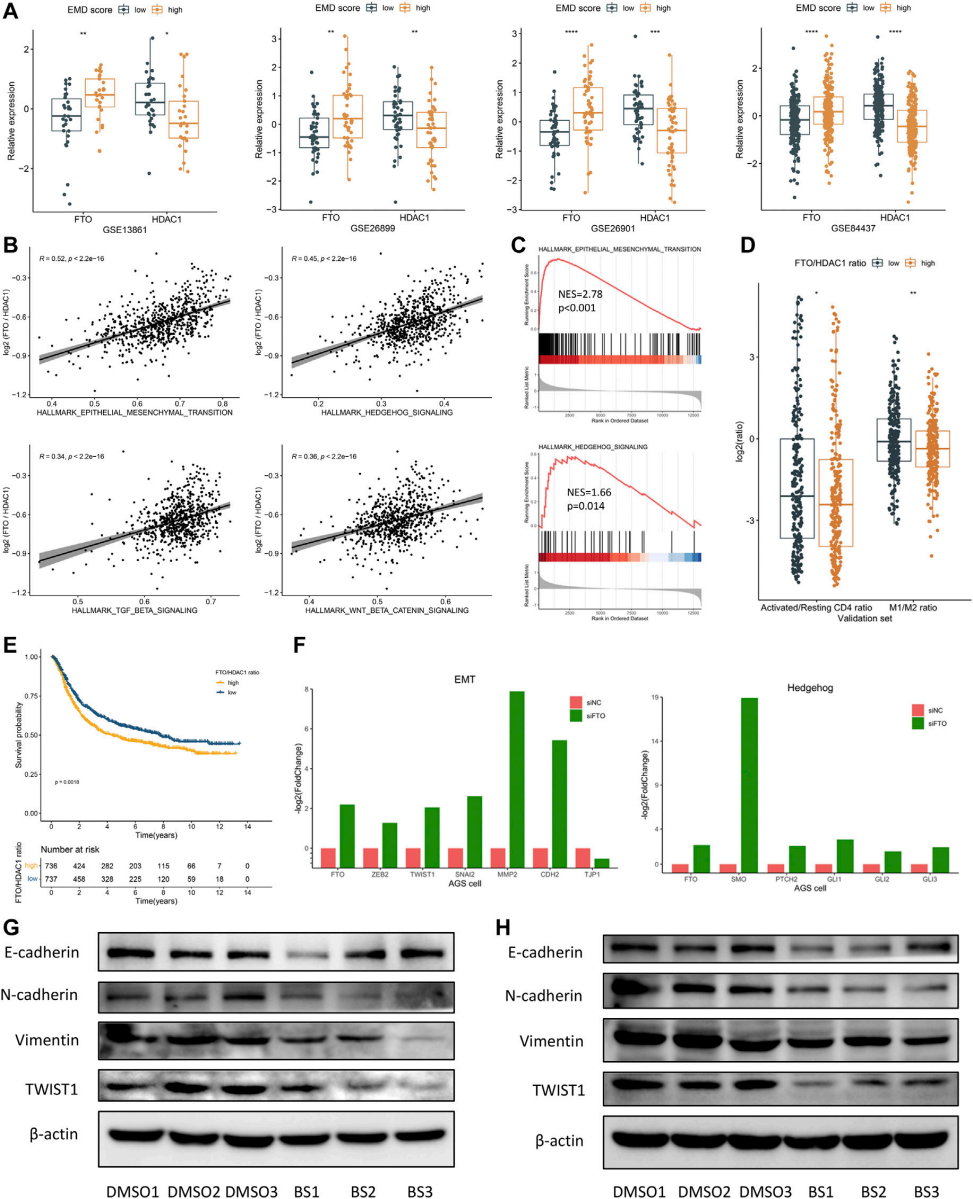
FTO may serve as a potential target for patients with a high EMD score. **(A)** Correlations of the EMD score with FTO and HDAC1 in each cohort of the validation set. **(B)** Correlations of the FTO/HDAC1 ratio with multiple pathways in the validation set. **(C)** GSEA analysis confirmed the activation of EMT and Hedgehog pathways in patients with a high FTO/HDAC1 ratio. **(D)** Correlations of the FTO/HDAC1 ratio with the activated/resting CD4^+^ T cell ratio and M1/M2 ratio in the validation set. Activated/resting CD4^+^ T cell ratio refers to the ratio of activated CD4^+^ T cells and resting CD4^+^ T cells. The M1/M2 ratio refers to the ratio of macrophage M1 and macrophage M2. **(E)** Kaplan–Meier curves showed the different overall survival between the high and low FTO/HDAC1 ratio group in the whole cohort of this study. Patients were separated into high and low groups according to the median EMD score of the whole cohort. **(F)** Barplots showed the fold change of EMT and Hedgehog markers under FTO depletion in the AGS cell line (GSE178697). **(G)** Western blot showed the suppression of the EMT pathway under FTO inhibition in the SNU719 cell line. **(H)** Western blot showed the suppression of the EMT pathway under FTO inhibition in the SGC7901 cell line (*p < 0.05, **p < 0.01, ***p < 0.001, and ****p < 0.0001).

Fat mass- and obesity-associated protein (FTO) was recently reported to participate in multiple tumorigenic processes including immune evasion in several malignant tumors (Li et al., 2017; Zhao et al., 2020; Tao et al., 2021; Zhou et al., 2021), implying the therapeutic potential of FTO inhibition in treating multiple cancers. In this study, high FTO expression and low HDAC1 expression were both observed in patients with high EMD score (Figure 8A). Moreover, FTO and HDAC1 were the most positively correlated (Spearman-r = 0.48, *p* < 0.001) and negatively correlated (Spearman-r = −0.45, *p* < 0.001) regulators relating to the EMD score, which also represented the m6A and histone modifications, respectively. To characterize the EMD subtype with both histone and m6A modification features, we measured the FTO/HDAC1 ratio for each GC patient. Interestingly, we found the FTO/HDAC1 ratio closely correlated with the EMD subtype (Supplementary Figure S4B), as well as the EMT (r = 0.52, *p* < 0.001, Figures 8B, C) and TGF-β (r = 0.34, *p* < 0.001, Figures 8B, C) pathway activation, which indicated stromal-activated TME characteristics. Also, a high FTO/HDAC1 ratio was accompanied by a low CD4^+^ T cell activation rate and a low M1/M2 ratio (Figure 8D), which indicated immune-suppressed TME characteristics. Moreover, a high FTO/HDAC1 ratio was related to a worse OS in the whole cohort of this study (Figure 8E). These findings revealed the close links of FTO with GC prognosis and multiple tumorigenic pathways.

These links were further evidenced by the significant downregulation of activated EMT markers (ZEB2, TWIST1, SNAI2, MMP2, and CDH2) in a GC cell line with FTO knockdown (FTO^−KD^) (Figure 8F; Supplementary Table S12). Consistently, EMT (*p* < 0.001) and TGF-β (*p* = 0.007) suppressions were also observed in an FTO^−KD^ colon cancer (CC) cell line (Supplementary Figures S3C–E; Supplementary Table S12). Targeting FTO, as we assumed, might help to improve the immune-suppressed TME caused by the activated EMT and TGF-β pathway.

Brequinar sodium (BS) was reported to bind tightly with FTO protein and inhibit the demethylase activity of FTO (Su et al., 2020). To confirm the FTO’s function of regulating the EMT pathway, we conducted *in vitro* experiments using two GC cell lines, SNU719 and SGC7901. Strikingly, when treated with BS, markers of EMT activation including N-cadherin, Vimentin, and TWIST1 were downregulated in both cell lines (Figures 8G, H). Although no significant upregulation of E-cadherin (the epithelial marker) was observed in the BS-treated cell lines, we still noticed a remarkable increase of the E-cadherin/N-cadherin ratio after BS treatment, especially in SGC7901 cell line (*p* < 0.05, not presented). These findings suggested FTO as a promising target for inhibiting the EMT pathway in GC, especially for patients with high EMD score.

## 4 Discussion

Extensive interactions between epigenetic modification types in shaping the TME of cancers have drawn increasing attention recently. Chen et al. systematically characterized the interactions of four RNA modification types (m6A, N1-methyladenosine, alternative polyadenylation, and adenosine-to-inosine RNA editing) and demonstrated that multilayer alterations of RNA modification “writer” are associated with patient survival and TME cell-infiltrating characteristics (Chen et al., 2021b). Zhao et al. focused on the relationship between LncRNAs and histone/DNA modifications and identified key LncRNA regulators as a prognostic biomarker for breast cancer subtypes (Zhao et al., 2021b). Similarly, regarding GC, Meng et al. (2021) elucidated the interactions between DNA methylation regulators and generated a DMS score for separating GC patients with distinctive prognosis and treatment efficacy (Meng et al., 2021). These studies provided insights into the active interaction networks of epigenetic modifications, suggesting their pivotal roles in shaping the TME across multiple tumors. However, the interactions between histone and RNA modifications and their impact on the TME in GC had not been fully explored yet.

In this study, for the first time, we comprehensively depicted the epigenetic regulation network including histone modification, RNA modification, and LncRNA in GC. Both in genome and transcriptome levels, we characterized the crosstalk between regulators of these four modification types. Zhan et al. found that several important histone regulator genes, including KMT2D, KMT2C, CREBBP, and EP300, are frequently altered in esophageal squamous cell carcinoma (Song et al., 2014). In our study, we further observed frequent co-mutations of these regulators, such as KMT2D-CREBBP, KMT2C-EP300, and CREBBP-EP300 in GC, which implied potential vulnerabilities based on epigenetic-related synthetic lethality in GC (Yang et al., 2019). Moreover, by identifying EMRLs using ChIP/MeRIP-seq data, our study added new evidence to the crosstalk between epigenetic modifications and LncRNA, which is also one of the key members of epigenetic regulation (Kan et al., 2021). Meng et al. defined a DMS score based on specific DNA methylation patterns to recognize GC patients with immune activation status and enhanced efficacy of immunotherapy (Meng et al., 2021). Consistent with Meng’s study, strong links between epigenetic modification patterns and the tumor environment were observed in GC, which in combination suggested epigenetic modification as a promising resource for developing new vulnerabilities for TME-targeting therapy.

With strong complexity and heterogeneity, GC presented disappointing results in most of the clinical trials on novel agents during the last decade (Serra et al., 2019). Thus, increasing molecular classification proposals were developed to characterize the molecular and clinical features of GC so as to optimize individualized diagnosis and treatment. ACRG classification is a globally accepted classification system of GC reported by the Asian Cancer Research Group in 2015, which divides GC into four subtypes: MSI, MSS/EMT, MSS/TM53 (+), and MSS/TP53 (−) (Cristescu et al., 2015). According to the GSE62254 cohort, the MSI subtype had the best prognosis with more than 60% patients of intestinal histotype, while the MSS/EMT subtype had the worst prognosis, over 80% of which were the diffuse histotype. However, the epigenetic modification patterns underlying the MSS/EMT subtype remain largely unexplored. In this study, we surprisingly found the EMD subtype we defined mostly overlapped with the MSS/EMT subtype (91.4%, Supplementary Table S6) in the GSE62254 cohort. Consistently, the EMD subtype had the worst overall survival and mostly consisted of diffuse histotype (80%, Supplementary Table S6). Our findings revealed the distinctive epigenetic modification patterns existing in the MSS/EMT subtype. By identifying the EMD subtype, our study provided not only new strategies for recognizing patients with poor prognosis in GC but also new sights into the epigenetic characterization of the widely used ACRG subtype.

Though with the rapid progress of ICI therapy in recent years, there were still limited GC patients benefiting from the ICI treatment. According to a meta-analysis based on 2003 patients from nine clinical trials, the objective response rate and disease control ratio were 9.9 and 33.3%, respectively, for advanced gastric or gastroesophageal junction (G/GEJ) cancer treated with ICI therapy (Chen et al., 2019). Mechanistically, the immune-suppressed TME may be one of the main causes of the resistance to ICI treatment. Hegde et al. proposed three distinct immunophenotypes (inflamed, immune-excluded, and immune-desert) based on the spatial distribution of CD8^+^ T cells in the TME (Hegde et al., 2016). Immune-excluded tumors were featured with an immune-suppressed TME, represented by T cells clearly embedded in the tumor stromal microenvironment with the presence of low MHC-I expression, TGF-β activation, myeloid inflammation, and angiogenesis (Hegde et al., 2016). Consistent with immune-excluded tumors, the EMD subtype we defined was characterized by the immune-suppressed TME with a low MHC-I expression, TGF-β activation, and angiogenesis activation, which also presented a low response rate to ICI treatment. Moreover, the EMD score we developed showed promising ability in recognizing GC patients with poor prognosis and resistance to ICI therapy. Desbois et al. reported that TGF-β-activated fibroblasts contribute to an immune-suppressed environment by cytokine production in ovarian cancer (Desbois et al., 2020; Desbois and Wang, 2021). In our study, TGF-β activation and high abundance of CAF were both observed in patients with a high EMD score, strongly implying the close links between TGF-β-activated fibroblasts and resistance to ICI treatment in GC.

Recently, combined therapy of ICI with radiotherapy, chemotherapy, or targeted therapy became a prospective strategy for improving the efficacy of cancer treatment. Results from CheckMate 649 showed that nivolumab with chemotherapy improved OS [hazard ratio (HR) 0.71 (98.4% CI 0.59–0.86); *p* < 0.0001] and PFS [HR 0.68 (98% CI 0.56–0.81); *p* < 0.0001] in GC patients with a PD-L1 CPS (combined positive score) of five or more when compared with chemotherapy alone (Janjigian et al., 2021). As one of the pivotal roles contributing to the immune-suppressed TME, the EMT pathway provided multiple therapeutic targets for combined therapy of ICI (Dongre et al., 2017; Jiang and Zhan, 2020). Combined ICI therapy with anti-EMT therapy would be a promising strategy, especially for patients with significant EMT activation. In our study, close correlations of the FTO overexpression with a high EMD score and activation of multiple tumorigenic pathways including EMT, TGF-β, and Hedgehog were observed. Moreover, a significant downregulation of the EMT pathway was observed in two FTO^−KD^ cell lines (Figure 8F, S1C-E), implying the potential links between FTO and the immune-suppressed TME. Su et al. demonstrated that FTO inhibition sensitizes leukemia cells to T cell cytotoxicity and overcomes hypomethylating agent-induced immune evasion (Su et al., 2020). Liu et al. developed a novel FTO inhibitor, Dac51, which can block FTO-mediated immune evasion and synergize with the checkpoint blockade for better tumor control (Liu et al., 2021). In this study, through the *in vitro* experiments of FTO inhibition conducted in two GC cell lines, we demonstrated that pharmacological inhibition of FTO significantly suppressed the EMT pathway. Our findings provided new clues of FTO’s participation in immune evasion and also suggested FTO as a potential target of combined ICI therapy with anti-EMT therapy in GC.

This study also has some limitations. First, the ability of the EMD score in predicting ICI efficacy needs to be further validated using more GC cohorts in the future. Besides, it remains unclear how FTO regulates the EMT pathway. Further studies are urgently needed to explore the underlying mechanism and verify the efficacy of combined ICI therapy with FTO inhibition in GC.

## 5 Conclusion

Collectively, we comprehensively characterized the crosstalk between histone and RNA modifications and identified the EMD subtype of GC with poor survival and distinctive TME characteristics. EMD score is a good indicator for prognosis and TME characteristics in GC and also might be a promising tool for recognizing patients suitable for combination ICI therapy.

## Data Availability

The datasets presented in this study can be found in online repositories. The names of the repository/repositories and accession number(s) can be found in the article/Supplementary Material.
