# Supplementary material for: Crosstalk of Histone and RNA Modifications Identified a Stromal-Activated Subtype with Poor Survival and Resistance to Immunotherapy in Gastric Cancer
Source: Front Pharmacol. 2022 May 5;13:868830. doi: 10.3389/fphar.2022.868830 (PMC9117636; doi:10.3389/fphar.2022.868830)
Supplement: Supplementary file 1 [file DataSheet1.docx]

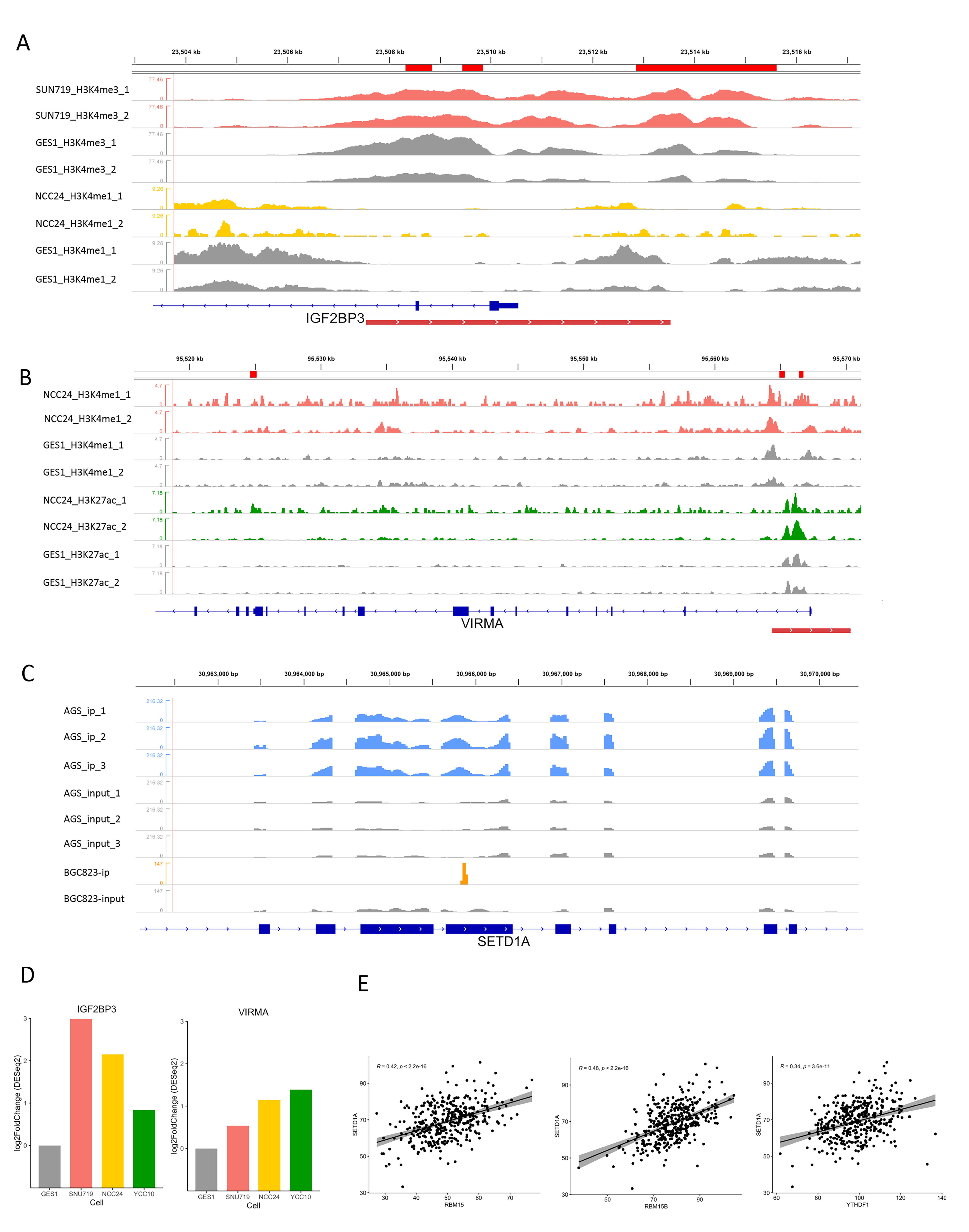


Figure S1 Supplementary figures of Figure 2. (A) Differential histone modifications in m6a regulators IGF2BP3. Peaks in red and yellow refer to the H3K4me3 and H3K4me1 modification in GC cell lines respectively, and grey refers to the corresponding modification in GES-1 as control. The red line at the bottom refers to the promoter region. (B) Differential histone modification in m6a regulators VIRMA. Peaks in red and green refer to the H3K4me1 and H3K27ac modification in GC cell lines respectively, and grey refers to the corresponding modification in GES-1 as control. The red line at the bottom refers to the promoter region. (C) Differential m6a modification in histone modification regulators SETD1A. Peaks in blue and orange refer to the immunoprecipitation (IP) signals of AGS and BGC823 cell lines respectively. Peaks in grey refer to the corresponding input signals. (D) Differential expression of IGF2BP3, and VIRMA between GC cell lines and GES-1 (p<0.05). (E) Correlations of SETD1A with the m6a regulators including RBM15, RBM15B, and YTHDF1.


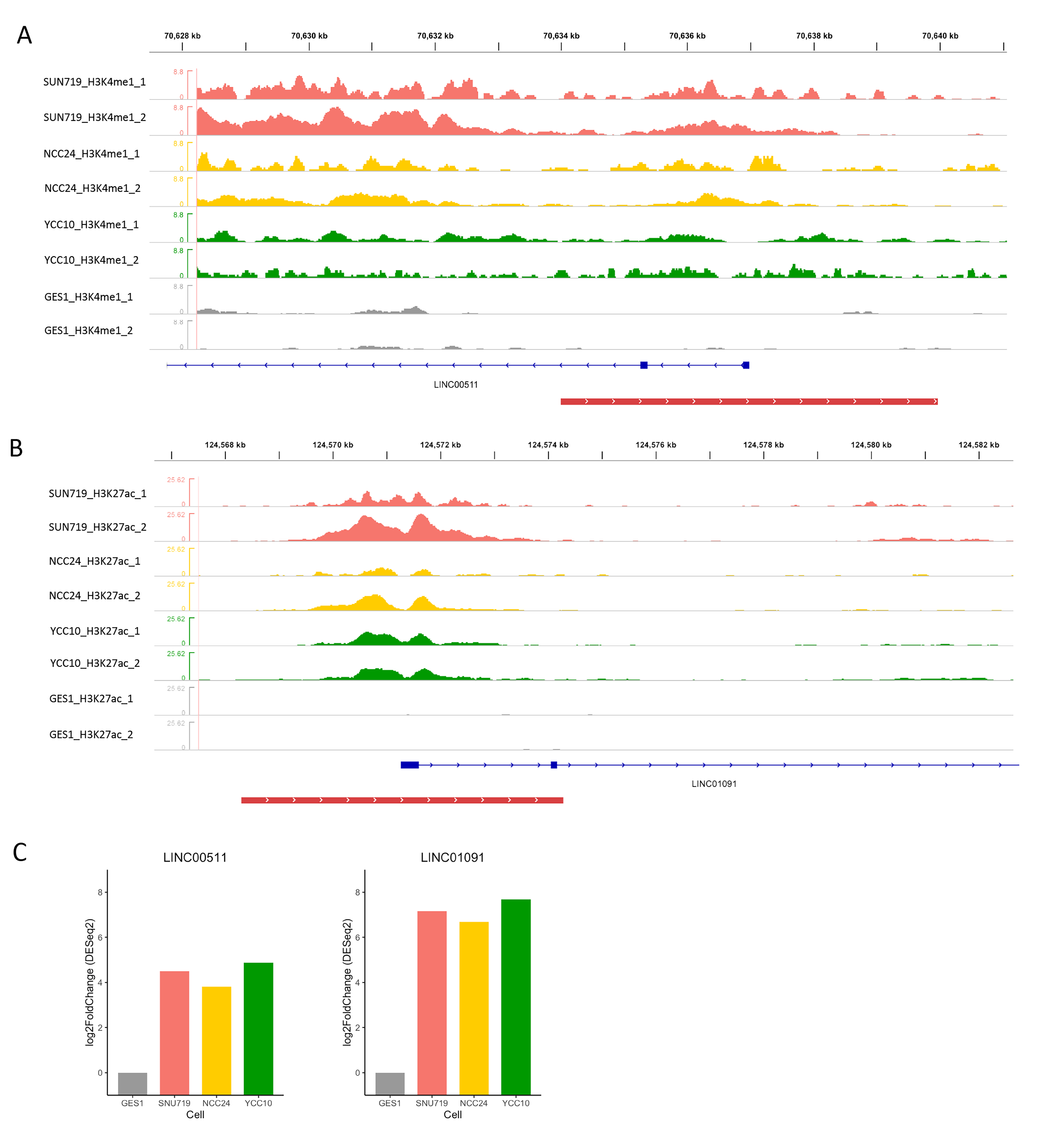


Figure S2 Supplementary figures of Figure 3. (A-B) Representative EMRLs (LINC00511, LINC01091) potentially regulated by the same histone modification type in multiple GC cell lines. Peaks in red, yellow, and green refer to the SNU719, NCC24, and YCC10 cell lines respectively, while grey ones refer to the corresponding modification in GES-1 as control. The red line at the bottom refers to the promoter region. (C) Differential expression of LINC00511 and LINC01091 in GC cell lines compared with GES-1 (p<0.05).


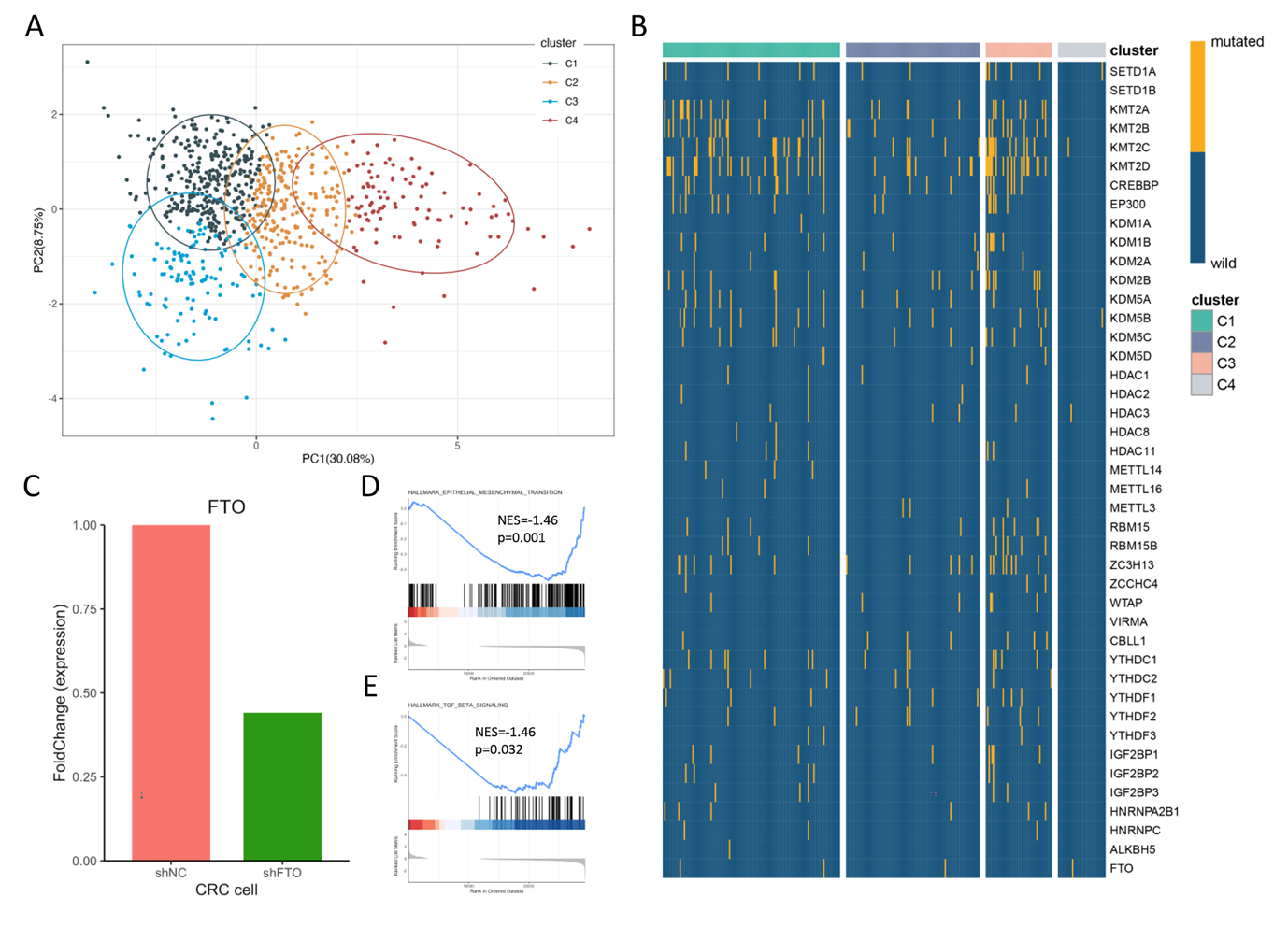


Figure S3 Supplementary figures of Figure 4 and Figure 8. (A) Principal Component Analysis (PCA) showed the heterogeneity of different clusters. (B) Heatmap showed the distinct mutation landscapes of the epigenetic regulators in the TCGA-STAD cohort. (C-E) GSEA analysis showed the suppression of the EMT and TGF-β pathways in a colon cancer celll line with FTO depletion (GSE165115).


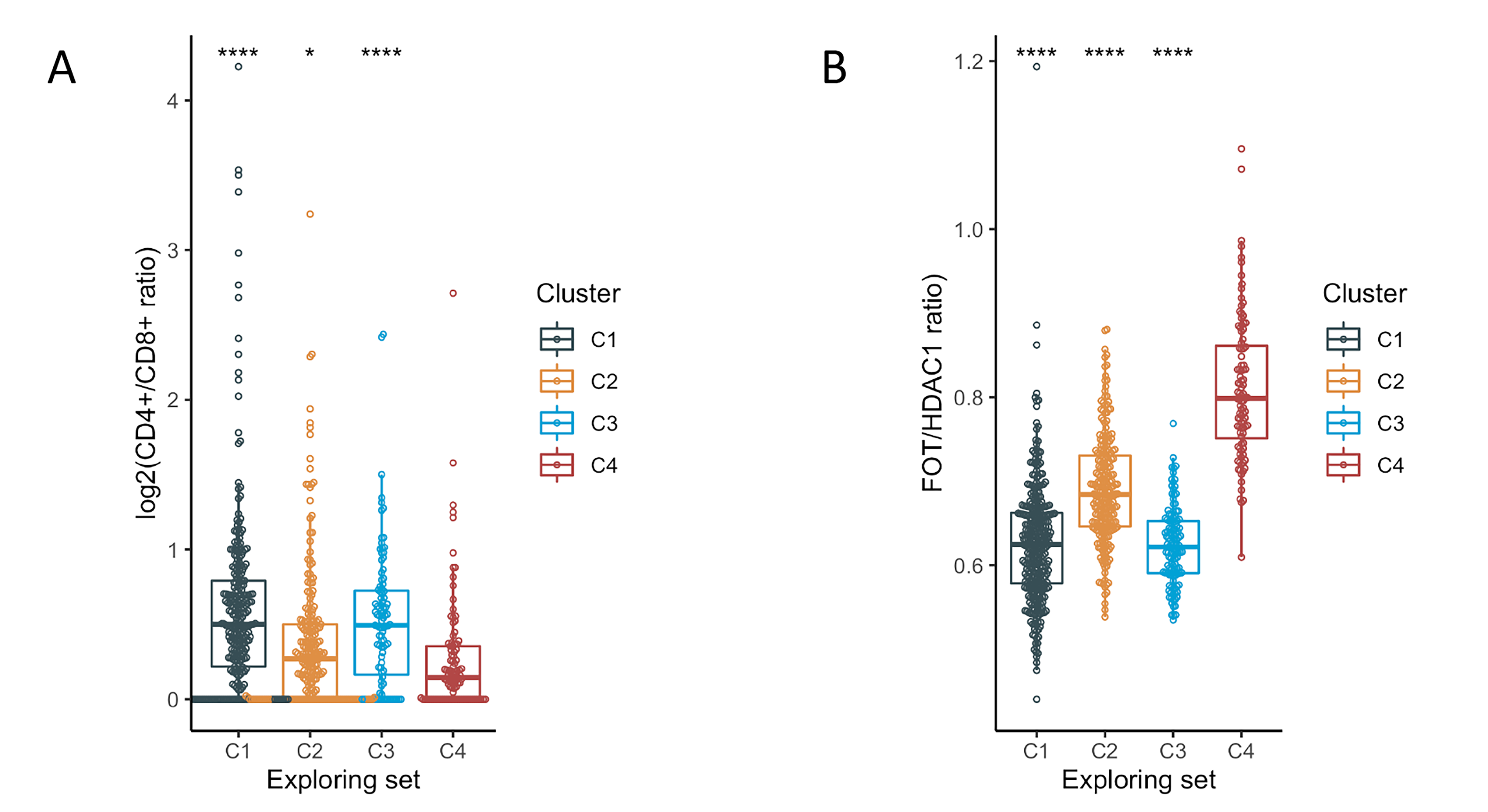


Figure S4 (A) Distribution of the CD4+/CD8+ T cell ratio among 4 clusters in the exploring set. (B) Distribution of the FTO/HDAC1 ratio among 4 clusters in the exploring set. ( *:p<0.05, **:p<0.01, ***:p<0.001, ****:p<0.0001)
